# Barriers to Medical Tourism Development in the United Arab Emirates (UAE)

**DOI:** 10.3390/ijerph18031365

**Published:** 2021-02-02

**Authors:** Iva Bulatovic, Katia Iankova

**Affiliations:** Faculty of Business, Higher Colleges of Technology, P.O. Box 41012, Abu Dhabi, United Arab Emirates; kiankova@hct.ac.ae

**Keywords:** medical tourism, development, barriers, United Arab Emirates, qualitative analysis

## Abstract

Medical tourism is a thriving industry. Many destinations now seek to attract more medical tourists. The United Arab Emirates (UAE) is no exception. As one of the most important pillars of the UAE’s economy, tourism is considered a high priority. However, medical tourism in the UAE is still developing. This paper addresses the main challenges for medical tourism in the UAE and proposes methods to enhance its development. This research utilized qualitative analysis. Twelve professionals in medical tourism were interviewed to provide data. The data were then analyzed using NVivo 12 software. Our results indicate that the key barriers to medical tourism development in the UAE are high costs of medical tourism services, lack of marketing activities, lack of collaboration between medical and tourism service providers, and so forth. Although initiatives from the UAE government are very encouraging, more efficient medical care supply networks, tourism suppliers and intermediaries should be established to ensure its growth. This research could influence national tourism policies in the UAE as well as regional alliances in all Gulf Cooperation Council (GCC) and the Middle East and North Africa (MENA) countries.

## 1. Introduction

Medical tourism is a thriving industry. The term “medical tourism” has been investigated for 20+ years [1,2]. Medical tourism can be defined as “the practice of travelling to another country with the purpose of obtaining health care (elective surgery, dental treatment, reproductive treatment, organ transplantation, medical checkups, etc.)” [3]. The most common procedures for medical tourists are as follows: cosmetic surgery, dentistry, and orthopedic treatments [4]. Medical tourists prefer a foreign country due to several reasons recognized by Karadayi-Usta and Bozdag [5]: “long waiting periods, high costs, the excessive number of patients, inadequate number of healthcare professionals and inadequate cutting-edge technological equipment at their country of residence” (p. 6475). In 2019, the medical tourism market was worth US $104,68 billion while forecasted to be US $273,72 billion by 2027 [6]. Currently, according to the Medical Tourism Index (MTI), the top destinations for medical tourism are Canada, Singapore, Japan, Spain, United Kingdom, Dubai, Costa Rica, Israel, Abu Dhabi, and India [7].

Tourism is an economic pillar for the United Arab Emirates (UAE). Indeed, the UAE actively positions itself within the tourism market as a destination for human-made wonders. In recent years, it has made significant improvements to expand tourist offerings and develop medical tourism in the country, particularly in the most tourism-developed emirates such as Dubai and Abu Dhabi. In 2016, the health strategy for Dubai 2021 [8] was approved by His Highness Sheikh Hamdan bin Mohammed bin Rashid Al Maktoum, Crown Prince of Dubai and Chairman of the Dubai Executive Council. This strategy proposed medical tourism development in the emirate with the mission of “transforming Dubai into a leading healthcare destination by fostering innovative and integrated care models and by enhancing community engagement” [8] (p. 10). As a result, a special entity (health tourism department) was formed—Dubai Health Experience (DXH)—with the aim to support medical tourism development the emirate and to attract more medical tourists. In terms of medical tourism, Dubai is most focused on cosmetic surgery, health check-ups, orthopedic surgeries, ophthalmology, dental treatments, dermatology, and fertility treatments [9]. In 2018, Dubai was visited by 337,011 medical tourists and generated a revenue of US $317 million [9]. DXH operates using a platform that connects 75 healthcare facilities, travel agents, and hoteliers. It also provides online consultations, reservations, insurance recommendations, and second medical opinion services and packages. In addition to Dubai, Abu Dhabi has also taken steps to increase its medical tourism. Its’ health strategy [10] also recognized the importance of medical tourism development in the emirate. Together, the Department of Health Abu Dhabi and the Department of Culture and Tourism—Abu Dhabi created a platform similar to DXH to increase medical tourism [9]. The platform connects 40 healthcare facilities and provides more than 280 medical treatment packages, insurance packages, appointments, hotels, recreational activities, and transport [9].

Although high-tech solutions to improve medical tourism have been implemented, the utilization of healthcare facilities could be improved further. In addition, in the UAE, medical tourism has yet to be developed outside of Dubai and Abu Dhabi. Areas such as Ras Al Khaimah and Al Ain could also benefit from medical tourism development, as well as other emirates such as Sharjah, Umm Al Quwain, Fujairah, and Ajman. The main purpose of this paper is to address the key barriers to medical tourism development in the UAE and to provide a recommendation for how the current situation could be improved in order to position the whole country among the top medical tourism destinations from the healthcare and tourism service quality perspective.

The following research questions were defined:

- What are the main barriers to medical tourism development in the UAE?
- What methods can be used to strengthen the development of medical tourism within the UAE?

By answering these two questions, the paper provides a clear overview of the medical tourism segments that should be improved. Moreover, the paper could help decision makers under the umbrella of medical tourism and at all levels to take concrete actions and overcome identified barriers.

## 2. Literature Review

Although medical tourism is a thriving industry, many countries have struggled to develop it successfully. Over the last two decades, medical tourism has been the focus of much research. However, the obstacles to medical tourism development in the UAE have yet to be explored using scientific qualitative analysis. This literature review presents an overview of existing research on critical factors that affect medical tourism development. 

### 2.1. Healthcare Service Quality and International Accreditation

The research conducted among medical tourism experts in Turkey says that one of the critical success factors for medical tourism development in the country is healthcare service quality. Actually, quality of healthcare is identified as one of top barriers to medical tourism development [11]. On the other side, similar research implemented in Africa among 36 managers of healthcare institutions in Russia says that quality plays a crucial role in medical tourism development [12]. From the patients’ perspective, healthcare service quality is the most important, and at the same time, it is the most critical factor of medical tourism development in the destination [13,14,15,16]. Apart from that, there are plenty of other studies that recognized healthcare quality as a vital motivator for international patients to travel abroad for medical purposes [17,18,19,20,21,22,23,24,25,26,27,28,29,30,31]. Al-Talabani and others [32] stated that the UAE lacks recognized doctors, which affects the quality of healthcare system. To ensure high quality of healthcare service and to attract more medical tourists, it is essential to obtain international accreditation [16,33,34,35,36,37,38]. This is particularly important for developing countries. According to Joint Commission International (JCI, Oak Brook IL, USA), the UAE has been ranked first in the world in terms of accredited healthcare facilities [39]. The UAE is working extremely hard to ensure that all hospitals will get international accreditation by 2021 [39,40,41,42]. It is important to emphasize the fact that Kamassi [43] raised a question about nonexistent international Islamic accreditation that can positively impact international Muslim patients. The Dubai Healthcare City Survey showed that patients chose Dubai because of the medical service quality [44]. According to the Medical Tourism Index, Dubai and Abu Dhabi are very well positioned on the medical tourism market in terms of service quality [7]. 

### 2.2. Healthcare Costs

Many studies have shown that the cost of the healthcare service plays an important role in choosing a medical tourism destination [5,35,45,46,47]. For instance, Aydin and Karamehmet [48] collected data on Turkey’s patients and medical tourism professionals using surveys. Their results indicated that the key factors that affect medical tourism development, according to professionals, are credibility of medical service providers, cultural distance and service quality, political and economic stability, regulations and legal frameworks, physical distance, and, in last place, healthcare cost. On the other side, patients identified healthcare costs as the most important factor influencing their decision to choose a destination for medical treatments. Moreover, Heung et al. [49] investigated barriers to medical tourism development in Hong Kong. They recognized high healthcare costs that result in high prices for customers as one of the crucial barriers to medical tourism development. Medical tourists in Thailand stressed high cost of healthcare as a key disadvantage of further development of medical tourism [50]. Jain and Ajmera also identified the high cost of medical services as a key barrier to medical tourism development in India [51]. On the other side, Al- Talabani and others stated that one of barriers to medical tourism development in Dubai is the high cost of medical treatments [32]. 

### 2.3. International Medical Insurance and Medical Tourism Infrastructure

International medical insurance is one of the key motivational factors for patients to choose one particular medical tourism destination and one of the stumbling blocks for destinations that intend to be medical tourism destinations [16,31,45,52]. Moreover, medical tourism infrastructure is also recognized as a vital factor for medical tourism development. Developing medical tourism could also be beneficial for the hotel industry. For example, high-class hotels in Greece have shown an interest in investing more in medical tourism due to its potential benefits [53]. On the other hand, a study showed that hotels in Malaysia may not be prepared for medical tourism due to environmental, organizational, and technological issues [54]. Kazemha and Dehkordi [55] defined a concept of hospital hotel as “a combination of a hotel as a resort and a hospital as a place of healing and rejuvenation that in addition to the course of treatment provides accommodations after treatment as well” (p. 515). The UAE is quite progressive in terms of healthcare infrastructure development [32]. However, hospital hotels have not been developed yet in the UAE. It is important to address that the number of hospital beds in the UAE has increased drastically in the last ten years. By the end of 2020, it is expected to have 14,000 hospital beds allocated across the UAE [39]. 

### 2.4. Medical Tourism Facilitators and Marketing

Mohamad et al. [56] stated that travel facilitators play a crucial role in developing medical tourism and suggest “tourism and travel facilitation and concierge services form a three-dimensional support system for health travellers” [56] (p. 362). Furthermore, Skountridaki [57] suggests that improved communication and cooperation between medical professionals and medical travel facilitators are required. Other studies confirm that lack of collaboration and networking among medical tourism suppliers and medical tourism facilitators is one of the key issues in medical tourism development [29,31,32,43,58,59,60,61,62,63]. Frederick and Gan [64] identified differences between Western and Eastern medical tourism facilitators. According to them, Eastern medical tourism facilitators promote a destination’s medical tourism services, while Western facilitators are mainly focused on advertising medical tourism services abroad. Tham [29] concluded that stakeholders, including medical tourism facilitators, should be more involved in planning and developing medical tourism destinations. On the other side, Kamassi [43] mentioned that above 50% of foreign patients have come to the UAE for medical treatment through medical tourism facilitators. 

Furthermore, lack of advertising g and digital marketing, and low internet utilization were also recognized [49]. Momeni et al., Amouzagar et al., and Mahdavi et.al [65,66,67] explored medical tourism development in Iran. They found that the main obstacles were, among others, a lack of marketing as well as poor branding and inferior management. Lee and Yudi [68] emphasized the importance of collaboration among medical tourism suppliers and media. Azimi et al., in their research, found out that word of mouth (WOM) is the most effective way to advertise medical tourism services according to the patients opinion [28,69]. Last, but not least, social media is increasingly utilized in medical tourism marketing, especially advertising [70,71,72]. 

### 2.5. Medical Tourism Strategic Management

Medical tourism development must be founded on a proper strategy and its’ proper implementation [45,49,59,73,74]. For example, a lack of policies and government actions was pointed out as the key barrier to medical tourism development in Hong Kong [49]. Furthermore, a lack of medical tourism standards was also recognized as one of barriers to medical tourism development in Yazd [45]. In the case of the UAE, Al- Talabani et al. identified lack of government policies that would involve the private sector in further investment in medical tourism development [32]. 

## 3. Methodology and Sample

While there are many qualitative research definitions, Merriam [75] suggests that qualitative research seeks to understand different meanings that people have constructed. In other words, qualitative research examines how people make sense of the world and their experiences. Qualitative research methods include interviews, observing participants, and textual analysis. In addition, Tracey [76] notes, “such methods can include research in the field, a focus—group room, an office or a classroom” (p. 29). In this research, an inductive thematic analysis approach was used. In other words, key themes were identified in relation to data [77,78] from interviews and mini focus group (panel discussion). As such, we should bear in mind that these themes may have “little relationship with the questions asked from the participants” [79] (p. 34). 

For the purpose of this research, semistructured in-depth interviews (IDI) were carried out with medical tourism professionals. This sampling type was homogenous, which is one of the most important premises for small samples [80]. In other words, the sample used in this research was similar in one or more dimensions [81]. In our case, each participant had over ten years of experience in the health industry and a special interest in tourism. The sample of 12 interviewees can be assumed as valid [79,82,83,84,85,86,87,88,89,90]. The snowball sampling method was used, which means that “the existing study subjects recruit future subjects among their acquaintances” [91] (p. 2). Saturation was discussed in line with Braun and Clarke’s recommendation [92], and based on consolidated criteria for reporting qualitative research (COREQ): a checklist and its 22nd item that is related to saturation [93]. Interviews were conducted from 20 October 2019 to the 31 January 2020. In-depth interviews were recorded and then analyzed using qualitative analysis (NVivo Pro 12). Due to the privacy policy adopted in this research, interview analyses are bias-free. A sample description is given in Table 1.

The questionnaire consisted of eight questions based on the literature review. An overview of the questions and their sources can be seen in Table 2.

### Mini Focus Group—Panel Discussion

This research also used data gathered from a mini focus group discussion held on 26 November at the Canadian University of Dubai in the format of public event called panel discussion. Mini focus groups usually comprise four or five participants [81]. In this research, the mini focus group contained four participants (Table 3).

The mini focus group was moderated by an experienced researcher, Dr Katia Iankova. The discussion was structured around five topics: hospital facilities for medical tourism, medical tourist expectations and satisfaction levels, competitors in neighboring countries, the role of medical tourism associations, and hospital accreditations for medical tourism. The discussion was attended by 22 academics, 10 practitioners, and 56 students.

## 4. Results and Discussion

### 4.1. Interview Results and Discussion

In this section, we present the interview results at a glance. We singled out and summarized the most significant answers of 12 respondents related to the key barriers to medical tourism development in the UAE. 

#### 4.1.1. Lack of High Healthcare Service Quality

Seven out of twelve respondents addressed healthcare service quality (clinical and customer service quality) as a key issue of medical tourism development in the UAE.

In line with that, Respondent 1 stated, “One of the biggest challenges for medical tourism development in the UAE is the lack of quality indicators. Then, incomplete medico-legal processes. Moreover, the lack of transparency in reports of clinical outcomes. Once these issues are sorted out, we can talk about high-quality medical services in the context of tourism development.” Respondent 2 said, “…What about quality of medical services? Resources’ capacity? Public transport capacity? Sponsorships and monitoring? All of these are problems for further medical tourism development in the UAE.” Respondent 3 indirectly mentioned lack of healthcare service quality through a real example: “They (doctors) are usually late which is a big issue from the patients’ satisfaction perspective. Follow up, control and monitoring must be improved.” Respondent 5 mentioned also lack of healthcare service quality: “Besides lack of quality and high prices of medical and hotel services I would say …” Respondent 8 referred to the lack of renowned doctors: “The biggest weakness is that the country does not offer quality (renowned) doctors in order to build a name of Medical destination.” Respondent 11 said, “Building credibility and trust with a patient for a high-quality service is an issue.” Respondent 12 said, “quality of medical services is questionable”.

These results correspond with studies of Daykhes et al. [12]; Hwang, Lee and Kang [13]; Jaapar et al. [14]; Javed and Ilyas [15]; John and Larke [16]; Al- Talabani et al. [32]. As stated before, healthcare service quality is essential for choosing a destination for medical treatments and procedures. These results are not surprising if we take into consideration the fact that interviewees were insiders, and they know what is going on “behind the scene”. However, if we see the rating of most developed medical tourism destinations in the UAE, such as Abu Dhabi and Dubai [7], these results are contradictory. It can be justified by the fact that the methodology of Medical Tourism Index (MTI) calculation is a bit different from ours. It is important to address that MTI is calculated based on an opinion survey conducted among Americans [7]. More importantly, according to available data, it is not noticeably clear whether those people were patients, medical tourists, or potential medical tourists. At the same time, validity, and relevance of the Medical Tourism Index in this case, is doubted by authors.

Besides healthcare service quality, it was mentioned that international accreditation of medical service providers plays a key role in medical tourism development and attracting medical tourists [16,33]. In that sense, interviewees did not mention that lack of international accreditation might be barrier to medical tourism development in the UAE. 

#### 4.1.2. High Costs and Prices of Medical Tourism Services

Interviewees identified high costs of medical tourism services as a key weakness and barrier to medical tourism development in the UAE. 

In line with that, Respondents 1 said, “Dubai is known as luxury destination, it is hard to expect that people will come for medical treatments in one of the most expensive destinations in the world.” On the other side, Respondent 4 mentioned that one of barriers to medical tourism development in the UAE is “high expenses of healthcare and accommodation”. Respondents 5 and 7 also identified high prices of medical and hotel services and cost of travel and accommodation as critical factors that affect medical tourism development in the UAE. Respondent 8 also mentioned that “the high prices of hotels, and flights are obstacles”. Respondent 10 stated that “the UAE is not accessible or affordable as medical tourism destination.” Lastly, Respondent 11 said that “high costs and packaging services along with the healthcare providers are also some of problems for better medical tourism development in the UAE”.

These results correspond with previous research on the topic [5,35,45,46,47]. If we compare our analysis to the analysis of Aydin and Karamehmet, it is noticeable that experts included in our research put high costs of medical tourism service first while experts in the above-mentioned study put high costs of medical tourism service last [48]. Additionally, Al–Talabani et al. [32] stated in conclusion that one of the issues that should be solved is high costs of healthcare services in Dubai. This conclusion corresponds with our analysis. 

#### 4.1.3. Lack of International Medical Insurance Accepted in the UAE

Almost all interviewees mentioned that one of the most critical barriers to further medical tourism development in the UAE is the lack of medical insurance coverage. 

Respondent 1 said, “There is no international insurance that is accepted in the UAE”. Respondent 4 discussed that “there is no or poor availability of international insurance coverage…” Respondent 5 as well as Respondent 6 also mentioned “Lack of overseas insurance coverage/lack of global insurance” as one of the key weaknesses/barriers to more dynamic medical tourism development in the UAE. Respondent 8 discussed in detail the key weaknesses of medical tourism in the UAE. The respondent mentioned “Another problem is that in many hospitals, certain insurance cards are not accepted (e.g., Adnic Gold is not accepted in top 1 hospital in Abu Dhabi—Cleveland Clinique).” Respondent 10 mentioned, “There is no insurance coverage in clinics or affordability of the medical visa.” Respondent 11 said, “Insurance (international) is a major challenge. Many services are not covered by health insurance.” Our results are in line with results published by John and Larke [16]; Abouhashem Abadi et al. [45], and Mandal [52]. 

#### 4.1.4. Lack of Medical Tourism Facilitators/Intermediaries and Marketing

Based on interviewees’ opinion, launching specialized travel agencies is a must for rapid improvement. Moreover, a collaboration between tourism and medical sectors is needed. 

Respondent 2 said, “People worldwide are not aware of the medical tourism in the UAE due to lack of advertising campaigns.” Respondent 5 said, “international patients are not aware of medical tourism here and what we have to offer”. Respondent 6 said, “I can sum up into lack of brand awareness and market orientation”. Respondent 8 mentioned, “The fractioned product and the lack of packages offered to the tourists. There are no, in my knowledge, specialized tour operators offering such packages including accommodation travel food, etc.” Respondent 10 said, “The UAE has a broad range of supporting infrastructure but no connecting entity, for example, the hotel and the clinic, and there is a lack of the communication between medical tourism suppliers.” Respondent 12 said, “There are many weaknesses of medical tourism in the UAE such as lack of advertising, there are no travel agencies specialized in medical tourism. Then, no collaboration between airports, airlines, tourism and hospitality sector and medical sector.” Our results confirm what has been proven in similar research conducted before by Mohamad et al. [56] and Skountridaki [57]. Facilitators in medical tourism have a vital role for further medical tourism development and its’ improvement in the UAE. In addition, interviewees identified a lack of advertising and destination branding as a medical tourism destination as one of the barriers for further medical tourism development. These results correspond with analysis provided by Momeni et al. [65]; Mahdavi et al. [67], and Heung et al. [67].

#### 4.1.5. Public Transport and Hot Weather

While some destinations struggle with accessibility and transport infrastructure, like Turkey [11], there are others that are assessed very well according to transport reliability, such as Penang [94]. In the case of the UAE, the key weakness is noticed in public transport, which is assessed by interviewees as underdeveloped. In other words, public connections between emirates are weak.

Respondent 1 said, “On the other side, there is **no developed public transport** on the destination, but infrastructure of medical tourism is very good”.

Respondent 2 also mention public transport and its capacity as a “problem” that must be fixed to ensure better medical tourism development in the UAE.

Respondent 4 discussed transportation barriers to medical tourism development in the UAE: “Dubai Airport—it is massive, it takes a lot of time to exit after disembarking. Road traffic is another challenge. Ambulance service is not necessarily the same brand as the destination hospital. Expensive aerial and terrestrial transport. Public transport is not well developed in terms of schedules. Taxies are becoming increasingly expensive (3 DRH 10 years ago and 12 DRH now as a starting price of the taxi before the counter starts). No intercity transport. No specialized equipped vehicles for transporting people with special needs (e.g., post-surgery) or if they exist are limited and extremely expensive”.

Moreover, interviewees recognized hot weather as one of obstacles for successful development of medical tourism. Respondent 4 discussed, “Also, the weather during summer is not convenient for medical tourism development in the UAE”. Respondent 6 said, “climate factors affect medical tourism development in the UAE”. Respondent 7 said, “The climate is too harsh.”

In the literature we reviewed, climate or weather conditions have not been emphasized as one of the critical factors that can affect medical tourism development in a destination or represented as one of the barriers to its development.

### 4.2. Mini Focus Group Results and Discussion

Mini focus group participants identified high costs and prices of medical and tourism services as a key weakness of medical tourism development in the UAE (mentioned by all four panelists), which is in line with previous research done by Wongkit and McKercher [50], and Mandal [52]. Fierce competition is another key threat to the development of medical tourism in the UAE. The following countries were identified as competitors: Turkey (was mentioned by Panelist 2), Thailand, Singapore, India (mentioned by all panelists), Iran (mentioned by Panelist 4), Balkan countries (mentioned by Panelist 2 and Panelist 4), and Russia (mentioned by panelist 1). Lack of high-quality procedures (mentioned by Panelist 2 and Panelist 3), insurance and rising costs (mentioned by all panelists) were also identified as key threats. Insurance appears as one of the vital legal barriers to medical tourism development in the UAE and is addressed by all panelists. These results are in line with results published in previous studies [16,31,45]. Panelists analyzed this issue from the residents’ point of view, too. They concluded that many expats based in the UAE have insufficient medical coverage. Instead, they travel to their home country for procedures, which corresponds with previous research done by John and Larke [16] and Mathijsen [23]. This is especially true for dentistry (mentioned by all panelists), fertility treatment (mentioned by Panelist 1 and Panelist 3), and illnesses susceptible to time extension to be treated (mentioned by all panelists). Panelists agreed that another important issue is the lack of specialized clinics, research centers, and internationally renowned doctors. These factors have already been identified by Al- Talabani et al. [32].

During the discussion, the lack of marketing and destination branding for the UAE as a medical tourism destination was discussed. No marketing campaigns for the UAE, past or present, have depicted the country as a medical tourism destination (mentioned by all panelists). Panelist 3 mentioned that tourism experts suggest that TV and social media adverts present “the UAE as a place for business, shopping, adventure and culture, but not for health purposes”. As discussed before [71,72], social media is not utilized enough for the purposes of medical tourism but represents an important communication channel between medical tourism supply and demand. Moreover, Panelist 4 mentioned that “during winter, the UAE’s warm weather could be marketed as a remedy for ‘winter blues’ and the ‘restorative’ mineral water in the hot springs of Ala Ain could also attract many medical tourists”, as stated by Panelist 2. Panelist 1 said that in 2018, the Dubai Health Experience (DHX) medical tourism hub was created. “The DXH portal enables people seeking treatments in Dubai to find a list of qualified hospitals and clinics in the relevant medical areas. However, most people are completely unaware that this service exists”, panelist 1 added.

Panelist 3 said: “What I see as the key problem are the lack of collaboration between hospitals and tourism stakeholders, high prices of accommodation and a lack of specialized medical tourist agencies, which equate to scarce and unreliable services.” Other panelists also confirmed that the lack of a strong network and collaboration between medical and tourism service providers might be even the most critical barrier to medical tourism development in the UAE. These results are in line with interview results, as well as with previous research [16,31,45,52].

On the other side, panelists stated that the medical institutions’ accreditation system in the UAE is favorable and there is no room for any drastic improvements, which confirms the Government’s ambitious intentions to ensure international accreditation for all hospitals by the end 2021 [40]. Last, but not least, management issues were identified, such as lack of human resources (mentioned by Panelist 1–3) that were mentioned also in research conducted by Cavmak and Cavmak [11], lack of leadership (mentioned by Panelist 3,4), lack of strategic planning [45,49], and a lack of collaboration between medical service providers and tourism stakeholders (mentioned by all panelists). These results correspond with previous research [45,56,57,63,73,74,95] and with interview results, too.

## 5. Conclusions

Based on our research, we can conclude that medical tourism is in the process of development only in two out of seven emirates. Dubai and Abu Dhabi have already been recognized as medical tourism destinations. Their local governments are also concentrated on medical tourism development. However, the rest of the country has not been explored yet in the context of medical tourism. According to the interview conducted among experts in medical tourism and mini focus group, panel discussion, we identified the key barriers to medical tourism development in the UAE, such as lack of high medical service quality, high costs (prices) of medical tourism services, lack of international medical insurance accepted in the country, lack of medical tourism facilitators, lack of advertising, lack of trust in the medical system by locals, lack of human resources, lack of transportation infrastructure, lack of strategic management, lack of collaboration between medical tourism stakeholders, and inconvenient weather conditions during summer. In line with the results, we recommend several steps that might help decision makers to improve medical tourism development in the UAE.

First of all, we recommend other researchers to confirm or deny our results by much more complex investigation, both qualitative and quantitative analysis, in order to propose directions on how the overall medical tourism development strategy should be designed. At this point, we assume that a top-down approach to medical tourism strategy development would be the most effective and efficient. Furthermore, in the process of crafting a medical tourism development strategy, representatives of medical and tourism sectors, as well as academia and residents, should be involved. We strongly believe that local medical tourism development strategies should be improved and/or developed by introducing high-quality medical tourism standards that will be equal for all destinations. Those standards might consider religious aspects also, which could attract more medical tourists who are Muslims. A particular focus should be on developing a robust system to ensure that international medical insurance will be accepted in the UAE. Pre-initial agreements between public (government) and private sectors (hospitals, clinics, and medical insurance companies) might work in this case. Once medical tourism strategies are defined, the set of medical tourism service quality indicators could be developed. This set of standards should cover all parties that have participated in medical tourism. On the other side, the whole process of customer/patient/medical tourist journey (from the beginning—booking, to the end—follow up, upon arrival of medical tourists in their home country) could be standardized, monitored, and controlled. The role of medical tourism facilitators could be clearly defined, as well as conditions under which the establishment of medical tourism facilitators’ companies (public/private) would be possible. Close collaboration between hospitals, clinics, hotels, tourism organizations, travel agencies, travel carriers, transportation companies, and medical and travel insurance companies is a must.

Secondly, to ensure the high quality of medical tourism in the UAE, it is vital to introduce long-term training programs for medical tourism participants, especially for hospitals’ and clinics’ managers, doctors, medical staff, and tourism stakeholders. This training could be proposed and delivered by the government; its departments dedicated to medical tourism development.

Thirdly, medical tourism products could be defined, as well as their prices. The government could propose instructions on how to design medical tourism products. We recommend three groups of medical tourism products: basic, silver, and gold. The basic package might include consultations with a doctor, checkups, medical treatment that is the main motive of travelling, flight tickets, accommodation, and transfers from airport to hospital/hotel and vice versa. The silver and gold medical tourism packages could be improved versions of the basic package with more additional services. In line with medical tourism product development, it is important to establish a special arrangement with air carriers in the UAE to ensure more affordable prices. For instance, defining special air tariffs for medical tourists and special services for medical tourists, such as qualified escorts, could work. Medical tourism services’ high costs/prices could be reduced by providing already prepared all-inclusive medical tourism packages.

Moreover, custom-made medical tourism packages also could be designed and offered to medical tourists. Again, here the role of medical tourism facilitators would arise. By creating medical tourism products/packages, the barrier—inconvenient weather conditions—could be eliminated, for example, by providing unique promotions for cosmetic treatments during summer without compromising the quality.

Fourthly, medical tourism in the UAE could be advertised more locally and overseas. Building trust, credibility, and the UAE’s reputation as a high-quality and safe medical tourism destination is needed. More specifically, the UAE has a reputation as a country that is safe and respectful towards everyone, but especially towards women and children. Medical tourists have the opportunity to combine medical treatments with other types of tourism, which could be an excellent selling point. This should be explored more and might become a key feature in advertisement campaigns. These campaigns should be initiated by government and private sector, as the public–private partnership (PPP) shows promising results in industries such as mega-events (e.g., Expo 2020).

Lastly, we have identified several limitations of the research: the topic was not investigated from the medical tourists’ point of view and the identified barriers were not explored in depth. We recommend researchers who would like to dig deep into the topic to pay attention to one particular barrier, do a thorough analysis and propose detailed solutions. Additionally, to apply any of the above strategic steps that we mentioned above, further study is needed, as we have already stated. To be precise, our research should be considered to be the very rough draft of guidelines on overcoming current barriers to medical tourism development in the UAE.

## Figures and Tables

**Table 1:** Interview Sample.

Participant	Job Title	Sector	Completion Date
Respondent 1	Head of Strategy	Public	1 November 2019
Respondent 2	CEO of Hospital	Private	28 October 2019
Respondent 3	Director of Health Insurance	Private	3 November 2019
Respondent 4	CEO of Hospital	Private	3 November 2019
Respondent 5	CO of Hospital	Private	28 October 2019
Respondent 6	CEO of Health Tourism company	Private	28 October 2019
Respondent 7	Medical Director and Cardiologist	Private	11 November 2019
Respondent 8	CEO of Hospital	Private	19 November 2019
Respondent 9	Supervisor, Health Risk Management	Private	8 January 2020
Respondent 10	Director of Marketing, Health Exchange	Public	8 January 2020
Respondent 11	Chair of Health Department (University)	Private	07 January 2020.
Respondent 12	Chair of Tourism Major (University)	Private	26 January 2020.

**Table 2:** Interview Questions.

Question	Sources
1. What are the weaknesses of medical tourism in the UAE?	[65,66,67]
2. What are the threats to medical tourism in the UAE?	[65,66,67]
3. What are the medical services provider challenges regarding medical tourists in the UAE?	[65,66,67]
4. What are the transport challenges of medical tourism development in the UAE?	[65,66,67]
5. What are the legal challenges for developing medical tourism in the UAE (visa, insurance)?	[65,66,67]
6. How would you assess the infrastructure of medical tourism in the UAE in general (hospitals, travel agencies, transport services, the readiness of hotels to accommodate medical tourists)?	[46,47]
7. What is a management (planning, organizing, leading, controlling) problem in this area?	[65,66,67]
8. What would you like to suggest or improve to enhance medical tourism in the UAE?	[46,47]

**Table 3:** Mini focus group sample.

Participant	Job Title	Sector
Panelist 1	Hospital CEO	Public
Panelist 2	Hospital CEO	Private
Panelist 3	Hospital CEO	Private
Panelist 4	Medical Tourism Company	Private

## Data Availability

Not applicable.
